# Blood Pressure and Spot Sign in Spontaneous Supratentorial Subcortical Intracerebral Hemorrhage

**DOI:** 10.1007/s12028-022-01485-4

**Published:** 2022-04-20

**Authors:** Joseph A. Falcone, Alex Lopez, Dana Stradling, Wengui Yu, Jefferson W. Chen

**Affiliations:** 1 Department of Neurosurgery, University of California Irvine, Orange, CA USA; 2 Department of Neurology, University of California Irvine, Orange, CA USA

**Keywords:** Cerebral hemorrhage, Hypertension, Blood pressure, Brain injury, Stroke, Computed tomography angiography

## Abstract

**Background:**

Spontaneous intracerebral hemorrhage is a potentially devastating cause of brain injury, often occurring secondary to hypertension. Contrast extravasation on computed tomography angiography (CTA), known as the spot sign, has been shown to predict hematoma expansion and worse outcomes. Although hypertension has been associated with an increased rate of the spot sign being present, the relationship between spot sign and blood pressure has not been fully explored.

**Methods:**

We retrospectively analyzed data from 134 patients (40 women and 94 men, mean age 62.3 ± 15.73 years) presenting to a tertiary academic medical center with spontaneous supratentorial subcortical intracerebral hemorrhage from 1/1/2018 to 1/4/2021.

**Results:**

A spot sign was demonstrated in images of 18 patients (13.43%) and correlated with a higher intracerebral hemorrhage score (2.61 ± 1.42 vs. 1.31 ± 1.25, *p* = 0.002), larger hematoma volume (53.49cm^3^ ± 32.08 vs. 23.45cm^3^ ± 25.65, *p* = 0.001), lower Glasgow Coma Scale on arrival (9.06 ± 4.56 vs. 11.74 ± 3.65, *p* = 0.027), increased risk of hematoma expansion (16.67% vs. 5.26%, *p* = 0.042), and need for surgical intervention (66.67% vs. 15.52%, *p* < 0.001). We did not see a correlation with age, sex, or underlying comorbidities. The presence of spot sign correlated with higher modified Rankin scores at discharge (4.94 ± 1.00 vs. 3.92 ± 1.64, *p* < 0.001). We saw significantly higher systolic blood pressure at the time of CTA in patients with a spot sign (184 mm Hg ± 43.11 vs. 153 mm Hg ± 36.99, *p* = 0.009) and the highest recorded blood pressure (*p* = 0.019), although not blood pressure on arrival (*p* = 0.081). Performing CTA early in the process of blood pressure lowering was associated with a spot sign (*p* < 0.001).

**Conclusions:**

The presence of spot sign correlates with larger hematomas, worse outcomes, and increased surgical intervention. There is a significant association between spot sign and systolic blood pressure at the time of CTA, with the highest systolic blood pressure being recorded prior to CTA. Although the role of intensive blood pressure management in spontaneous intracerebral hemorrhage remains a subject of debate, patients with a spot sign may be a subgroup that could benefit from this.

## Introduction

Spontaneous intracerebral hemorrhage (ICH) is a common cause of brain injury, accounting for 15–30% of strokes globally, and is associated with high rates of morbidity and mortality [1, 2] and a heavy socioeconomic burden on health care systems [3].

Hematoma expansion occurs in 20–30% of ICH cases [4–7] and leads to worse functional outcomes and increased mortality [4, 8–10]. Attempts to predict hematoma expansion often focus on radiographic features on computed tomography (CT) and CT angiography (CTA). Notable among these is the CTA spot sign, an enhancing focus of contrast extravasation in the setting of actively bleeding ICH first described in 1999 [8, 11]. This is seen in 20–50% of ICH cases [6–8, 12–18] and is a rapidly identifiable finding with reasonable interobserver agreement [6, 19]. It has consistently been shown to predict hematoma expansion [6, 11–15, 18, 20].

Predictors of outcome in ICH have also been a focus of research, and currently the ICH score is the most widely used prognosticator of short-term mortality [12, 21, 22]. The presence of the spot sign also correlates with poor functional outcome and increased mortality [6, 8, 11, 12, 14, 16–18, 23, 24], and understanding the factors contributing to the spot sign may provide insight to potential interventions.

The role of elevated blood pressure (BP) in the progression of ICH and the impact of intensive BP management remains an area of ongoing investigation [25]. The intensive blood pressure reduction in acute cerebral haemorrhage trial 2 (INTERACT-2) [26] compared intensive BP lowering with standard care (systolic blood pressure [SBP] < 140 mm Hg vs. < 180 mm Hg). It showed no increase in adverse events in the aggressive treatment group. There was no significant difference in mortality or disability at 90 days, although an ordinal analysis of modified Rankin scores showed improved functional outcomes with intensive BP lowering. Additionally, a meta-analysis suggests that intensive BP reduction was associated with a trend for lower risk of significant ICH expansion compared with standard treatment (odds ratio 0.82, 95% confidence interval [CI] 0.68–1.00, *p* = 0.056), especially in larger randomized controlled trials [27]. The 2015 American Heart Association ICH guidelines [28] have recommended that for patients with ICH presenting with SBP between 150 and 220 mm Hg and without contraindication to acute BP treatment, the acute lowering of SBP to 140 mm Hg is safe (Class I; Level of Evidence A) and can be effective for improving functional outcome (Class IIa; Level of Evidence B). Although hypertension is the cause of at least half of all ICH [29], the impact of elevated BP on the presence of the spot sign has yet to be fully clarified.

This investigation aims to explore the relationships between various BP parameters, the rate of BP control, the timing of CTA, and the presence of the spot sign. This is the first investigation, that we are aware of, to look at BP specifically at the time of obtaining CTA and its correlation to the spot sign.

## Methods

### Study Design and Settings

This is a single-center retrospective observational cohort study of patients admitted to a US tertiary academic medical center between 1/1/2018 and 1/4/2021. The protocol was approved by the center’s institutional review board, with waiver of informed consent. The data supporting the findings of this study are available from the corresponding author upon reasonable request.

### Patient Population

Adult patients were prospectively enrolled in the American Heart Association’s Get with The Guidelines-Stroke registry using the American Heart Association’s Patient Management Tool. This database was queried for patients with a diagnosis of ICH. To isolate spontaneous subcortical supratentorial hemorrhages, we set exclusion criteria including infratentorial origin, isolated intraventricular hemorrhage, hemorrhage isolated to the cortex without subcortical component, multifocal discontinuous hemorrhages, and hemorrhagic conversion of ischemic strokes. The decision to exclude cortical hemorrhages was made to focus this study on potentially hypertensive hemorrhages. Of note, lobar subcortical hemorrhages were included. This study was limited to patients with supratentorial hemorrhages to avoid potential confounders that the posterior fossa may introduce, including smaller volume for hematoma expansion and the clinically unique entity of brainstem hemorrhages.

We also excluded hemorrhagic tumors, cavernomas, infectious processes, calcifications, hemorrhage due to trauma, and ICH determined by CTA to be secondary to vascular lesions including aneurysms, arteriovenous malformations and dural arteriovenous fistula. We then excluded patients who did not have CTA performed within 24 h of presentation.

Patients who presented as “Stroke Codes” underwent prompt initial assessment by senior neurology residents. This protocol, initiated by the emergency department (ED) physician in any patient with acute onset neurologic deficit or altered mental status, involves initial assessment by the stroke neurology team, and rapid imaging with noncontrasted head CT as well as CTA to evaluate for intracranial hemorrhage or large vessel occlusion. Patients who did not present as a “Stroke Code” had CTA ordered at the discretion of the managing neurosurgeon or ED physician with concern for an intracranial pathological condition. As per our institutional practice, regardless of presenting protocol, hypertension was treated with a goal of rapidly achieving SBP < 140 mm Hg after diagnosis of ICH using a combination of intravenous pushes of labetalol (10 mg) or hydralazine (10 mg) in combination with continuous intravenous nicardipine infusion (5–15 mg/h).

### Outcomes

Primary objectives of this study included BP at various time points relative to arrival, symptom onset, and CTA imaging, and their relation to the incidence of spot sign.

BP measurements were made with digital sphygmomanometer by ED nursing staff on arrival and repeated at 5–15-min intervals until SBP < 140 mm Hg was achieved. Intraarterial BP monitoring was not routinely used prior to achieving the target SBP. BP data were retrospectively collected at time points including arrival, closest measurement to time of CTA, and highest BP recorded in the ED. Mean arterial pressure (MAP) was extrapolated as per preprogrammed algorithm from SBP and diastolic BP. To assess the rate of BP control, we calculated the duration from initial presentation to the first BP reading under 140 mm Hg. To assess the importance of fluctuating BP, we determined which patients had fluctuations in BP with SBP rising to 160–180 mm Hg or > 180 mm Hg after initially attaining goal of SBP < 140 mm Hg and specifically analyzed these cohorts.

The time of initial presentation was estimated by the earliest recorded information in the chart. The time of last known well (LKW) was estimated from reported history when this information was available. Those patients for which this information was not recorded were excluded from analysis involving this metric. The time of CTA was recorded and the intervals between initial presentation and CTA as well as LKW to CTA was calculated.

Secondary objectives, including patient demographics such as age, sex, comorbidities, laboratory results, vital signs and clinical information including ICH score, hematoma expansion, and outcomes, were collected by retrospective review of the electronic medical record.

Characteristics of the hemorrhage were collected including ICH score, ICH volume, presence of intraventricular hemorrhage, and presence of a spot sign. ICH volume was estimated by the AxBxC/2 method, which has been validated as a reliable approximation [30]. ICH volumes were calculated independently by two authors (JF and AL) and values were averaged to obtain final volume used in analysis. Cases with variability in measurement greater than 10 cm^3^ were additionally reviewed by JC, and the final measurement was reached by consensus between the three authors. Spot sign was defined by the presence of contrast extravasation within an acute hematoma with a minimum diameter of 1.5 mm in any dimension, either serpiginous or spot-like, without any connection with normal surrounding vasculature or corresponding hyperdensity on CT, and with attenuation at least double that of the background hematoma in Hounsfield units. This definition was validated in the Prediction of Hematoma Growth and Outcome in Patients with Intracerebral Hemorrhage Using the CT Angiography Spot Sign study [17]. Hemorrhage progression was defined here as increase in hematoma volume by > 33%, as established in the literature [31]. Indication for operative intervention, such as mass effect or low Glasgow Coma Scale (GCS), was at the discretion of the managing neurosurgeon, as was the operative approach.

Clinical data including GCS on presentation, length of stay in intensive care unit, and overall hospital stay, and in-hospital death were retrospectively collected. Functional outcome at time of discharge including Glasgow Outcome Score (GOS) and modified Rankin Score (MRS) were retrospectively determined based on description by managing physicians and, when available, assessment by physical therapist. Analysis of longer term outcomes was attempted via retrospective chart review but was precluded by overall low rates of follow up.

### Statistics

Student’s *t*-test was used to compare data for continuous variables between the two groups, assuming unequal variance. *χ*^2^ test of independence was used to compare data for noncontinuous and categorical variables. A *p* value < 0.05 was considered statistically significant. A separate analysis was performed of patients with available information on LKW to assess the relative influence of timing of CTA and BP at time of CTA using univariate and multivariate binary logistic regression analysis. IBM SPSS Statistics (Version 28.0; IBM Corp, Armonk, NY) was used for the statistical analysis.

## Results

### Incidence of Spot Sign

**Fig. 1:**
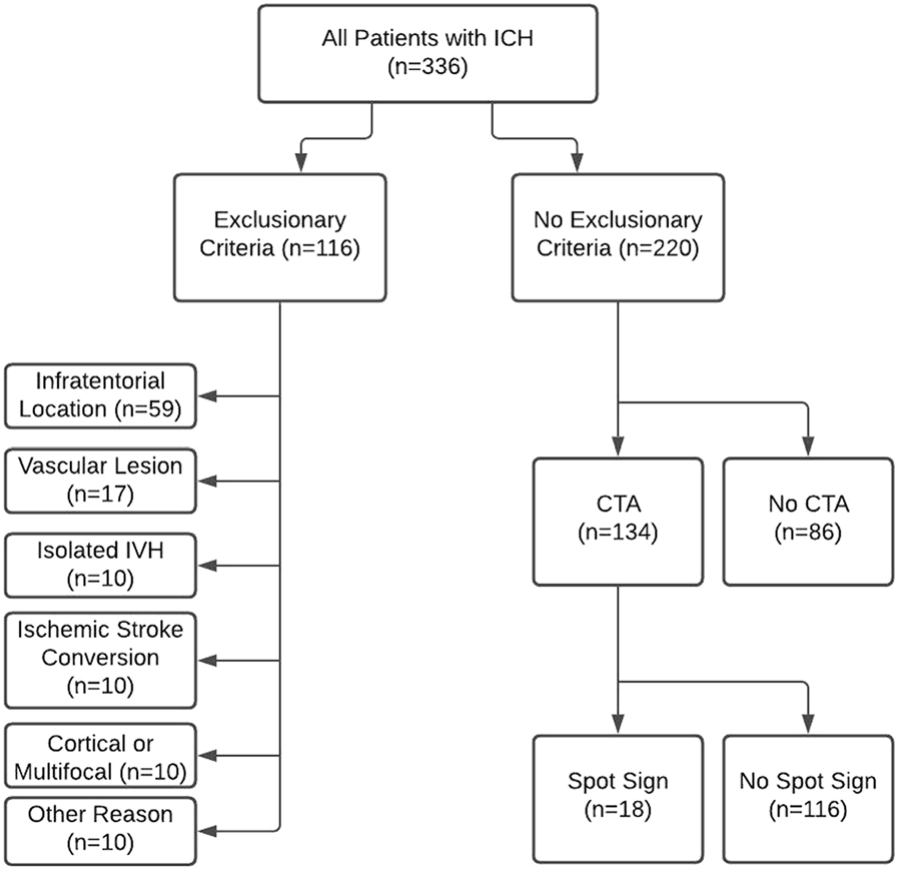
Breakdown of patient cohorts. A total of 336 patients were identified with a diagnosis of spontaneous intracerebral hemorrhage (ICH). A total of 116 were excluded from analysis on the basis of clinical and radiographic criteria. A total of 86 were excluded because of the absence of CTA within 24 h of presentation. A total of 134 were included in final analysis, of whom 18 had a spot sign on CTA and 116 did not. *CTA* computed tomography angiography, *IVH* intraventricular hemorrhage

**Fig. 2:**
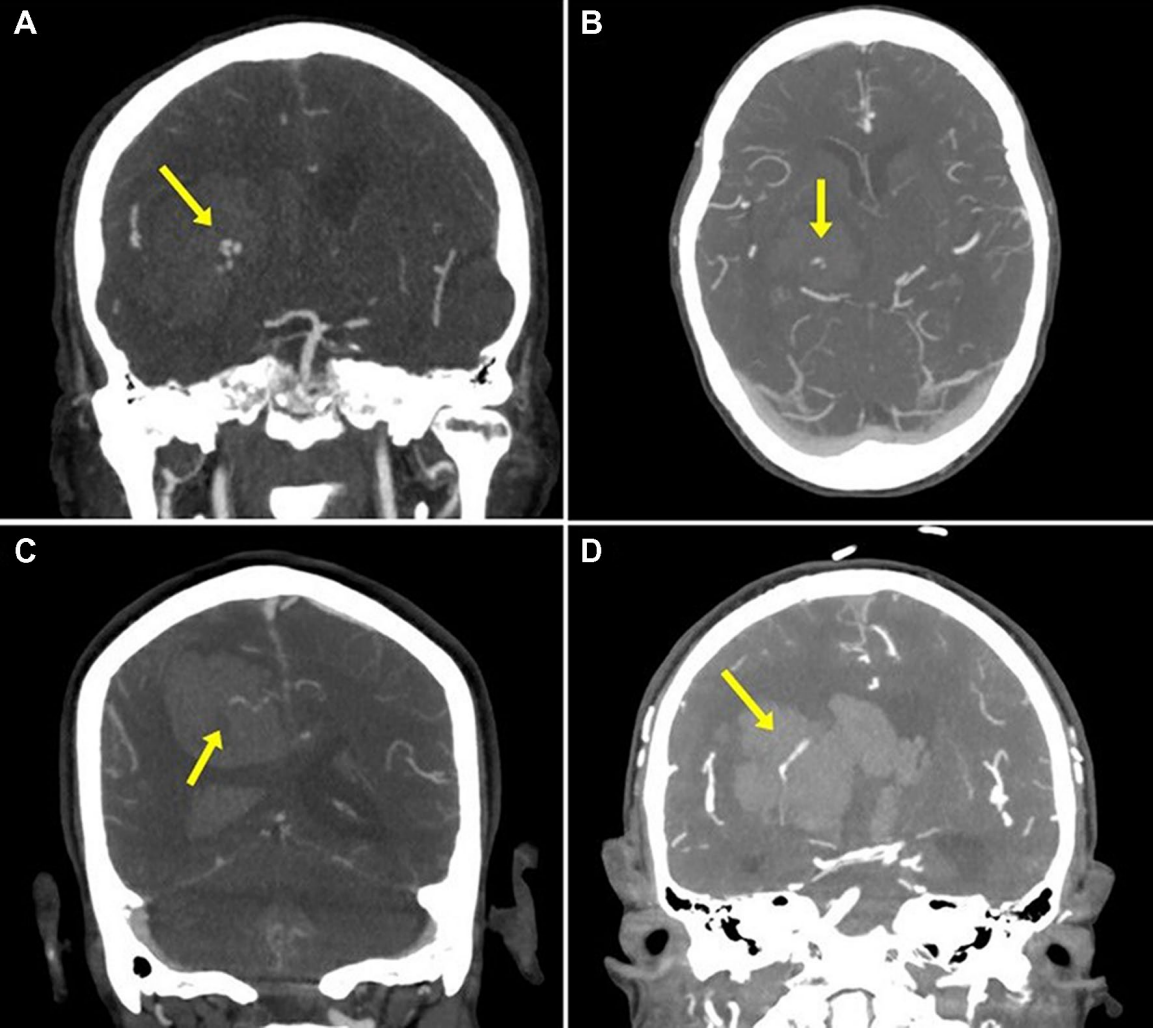
Computed tomography angiograms demonstrating examples of spot sign (yellow arrows) in four different patients. The appearance of the spot sign was variable, including examples with multiple associated foci of hemorrhage (**a**), small, isolated foci of hemorrhage (**b**), as well as streaks of extravasation appearing to come from branches of the anterior cerebral artery (**c**) or the branches of the middle cerebral artery, with lenticulostriate arteries particularly being a frequent apparent source of extravasation (**d**)

A total of 336 patients were identified in the database with a diagnosis of ICH between 1/1/2018 and 1/4/2021. Of these, 116 met our predefined exclusionary criteria, as detailed in Fig. 1. Of the 220 remaining patients, a total of *n* = 134 had a CTA performed within 24 h of arrival and were included in the final analysis. Of these, 18 (13.43%) were seen to have a spot sign on CTA, and 116 (86.56%) were not. Figure 2 demonstrates characteristic examples of a spot sign on CTA.

### Patient Characteristics

Baseline comorbidities and mean laboratory values on arrival for patients with and without a spot sign are presented in Table 1. Significant differences in mean ICH scores and several component metrics were seen between these groups and are presented in Table 2. The intraclass correlation coefficient between the two initial raters (JF and AL) for assessment of volumes was 0.913 (95% confidence interval [CI] 0.880–0.937). All patients except for two (both without a spot sign on CTA) had at least one delayed CT to evaluate for hematoma expansion, and there was a greater percentage of patients with a spot sign who demonstrated hematoma expansion (16.67% vs. 5.26%, *p* = 0.042).

**Table 1:** Comparison of baseline comorbidities and serum laboratory results between patients with and without a spot sign

Parameter	Spot sign, *n* (%)	No spot sign, *n* (%)	*p* value
Age	62.39 (16.81)	62.13 (15.63)	0.948
Male sex	12 (66.67)	82 (70.69)	0.729
Stroke code	11 (61.11)	74 (63.79)	0.836
Undoctored	4 (22.22)	23 (19.83)	0.814
Anticoagulation	6 (33.33)	34 (29.31)	0.729
Prior hypertension	10 (55.56)	75 (64.66)	0.456
Prior hyperlipidemia	3 (16.67)	28 (24.14)	0.484
Prior diabetes	2 (11.11)	33 (28.45)	0.119
Prior CAD	1 (5.56)	11 (9.48)	0.587

Parameter	Spot sign, mean (SD)	Spot sign, mean (SD)	*p* value
Na	136.17 (3.87)	137.09 (3.78)	0.341
Glucose	181.56 (80.61)	158.16 (68.15)	0.189
Creatinine	0.97 (0.32)	1.38 (1.62)	0.287
WBC	8.93 (4.98)	10.89 (4.51)	0.094
Hgb	13.65 (2.18)	14.02 (2.09)	0.484
Platelet	192 (70.513)	231.90 (85.74)	0.063
INR	1.20 (0.40)	1.14 (1.08)	0.821

Data are presented as *n* (%) for categorical variables or mean (SD) for continuous variables

*CAD* coronary artery disease, *Hgb* hemoglobin, *IFT* interfacility transfer, *INR* international normalized ratio, *Na* serum sodium, *SD* standard deviation, *WBC* white blood cell count

**Table 2:** Comparison between patients with and without a spot sign’s mean ICH scores and component variables, as well as SBP and MAP at time of arrival, time of obtaining CTA, and overall highest recording while in the emergency department

Parameter	Spot sign, mean (SD)	No spot sign, mean (SD)	*p* value
ICH score	2.61 (1.42)	1.34 (1.25)	0.002*
GCS on arrival	9.06 (4.56)	11.74 (3.65)	0.027*
ICH volume (cm^3^)	58.37 (38.75)	25.13 (27.88)	0.001*
Age (yr)	62.39 (16.81)	62.13 (15.63)	0.952

*CTA* computed tomography angiography, *GCS* Glasgow Outcome Score, *ICH* intracerebral hemorrhage, *MAP* mean arterial pressure, *SBP* systolic blood pressure

*Indicates statistical significance

### Association with BP

A total of 8.95% of patients underwent placement of an arterial line prior to achieving goal SBP < 140 mm Hg. Overall, 5.97% of patients were managed with prn medications alone, 5.22% were managed with continuous infusion alone, and 88.8% were managed with a combination of the two.

BP at multiple time points significantly correlated with the presence of a spot sign and are detailed in Table 3. Greatest significance was seen for SBP at the time of CTA (184 mm Hg [standard deviation {SD} 43.11] vs. 153 mm Hg [SD 36.99], *p* = 0.009), although the highest SBP recorded (217 mm Hg [SD 40.92] vs. 191 mm Hg [SD 37.00] *p* = 0.019) was also significant.

**Table 3:** Comparison between patients with and without a spot sign of SBP and MAP at time of arrival, time of obtaining CTA, and overall highest recording while in the emergency department

Parameter	Spot sign, mean (SD) (mm Hg)	No spot sign, mean (SD) (mm Hg)	*p* value
SBP on arrival	200.72 (48.04)	179.01 (37.18)	0.081
MAP on arrival	136.87 (33.53)	128.20 (47.56)	0.346
SBP at time of CTA	184.61 (43.11)	153.68 (36.99)	0.009*
MAP at time of CTA	124.91 (25.40)	106.80 (26.99)	0.010*
Highest SBP	217.33 (40.92)	191.36 (37.00)	0.019*
Highest MAP	146.78 (34.40)	131.27 (25.44)	0.081

*CTA* computed tomography angiogram, *MAP* mean arterial pressure, *SBP* systolic blood pressure

*Indicates statistical significance

It was seen that patients with a spot sign had a shorter duration between arrival and obtaining CTA (23.06 min [SD 32.02] vs. 176.76 min [SD 310.25], *p* < 0.001). Data on LKW were available for 72.22% of patients with a spot sign and 75.86% of patients without a spot sign. Among these patients, the duration from LKW to CTA was significantly shorter in patients with a spot sign (133.62 min [SD 110.15] vs. 1578.73 min [SD 4188.76], *p* = 0.002). Rate of BP lowering was assessed by the interval from arrival to the first SBP recording less than 140 mm Hg. This was statistically shorter in the spot sign group (53.11 min [SD 24.93] vs. 84.30 min [SD 116.93], *p* = 0.013). Likewise, patients with a spot sign obtained CTA earlier while obtaining BP control (time of CTA − time of SBP < 140 was − 30.06 min [SD 33.52] vs. 92.46 min [SD 313.42], *p* < 0.001).

After achieving initial goal SBP < 140, SBP was seen to fluctuate to 160–180 mm Hg in 41 patients (30.6%), and > 180 mm Hg in 19 patients (14.18%) within the first 24 h. No statistical significance was seen compared with those patients who had no major fluctuation after achieving goal SBP < 140 mm Hg and these groups for incidence of spot sign, hematoma expansion, or outcomes including discharge GOS or MRS (Table 4). Of these patients, nine had BP fluctuations after achieving SBP < 140 mm Hg and prior to the CTA, and no significant correlation was seen for this group with the incidence of spot sign (*p* = 0.221).

**Table 4:** Comparison between patients with lability of SBP after the initial goal of SBP < 140 mm Hg is achieved

Parameter	*n* (%)	*p* value
*Spot sign incidence*		
SBP < 160	10 (13.5)	
SBP 160–180	5 (12.2)	0.841
SBP > 180	3 (15.8)	0.799
*Hematoma expansion*		
SBP < 160	4 (5.5)	
SBP 160–180	2 (5.0)	0.913
SBP > 180	2 (10.5)	0.427

Parameter	Mean ± SD	*p* value
GOS at discharge		
SBP < 160	3.03 ± 1.146	
SBP 160–180	2.95 ± 1.161	0.736
SBP > 180	2.74 ± 1.368	0.347
MRS at discharge		
SBP < 160	3.93 ± 1.625	
SBP 160–180	4.2 ± 1.569	0.402
SBP > 180	4.26 ± 1.628	0.431

Outcomes are compared between patients whose SBP remained < 160 mm Hg, those whose SBP reached 160–180 mm Hg, and those with a SBP reaching > 180 mm Hg for incidence of a spot sign, hematoma expansion, and GOS and MRS at discharge

*CTA* computed tomography angiogram, *GOS* Glascow Outcome Score, *MAP* mean arterial pressure, *MRS* modified Rankin Scale, *SBP* systolic blood pressure

Binary logistic regression analysis of the incidence of spot sign with SBP at the time of CTA and the duration from LKW to CTA was performed to assess possible confounding between these variables. Significance for both duration of LKW to CTA (*p* = 0.015 [95% CI 0.988–0.999]) and SBP at CTA (*p* = 0.028 [95% CI 1.002–1.031]) was seen on univariate analysis, however in the multivariate model, duration from LKW to CTA remained significantly correlated with spot sign (*p* = 0.023 [95% CI 0.988–0.999]), whereas SBP at CTA did not (*p* = 0.660 [95% CI 0.986–1.022]).

### Outcomes

Patients with a spot sign showed a trend toward longer length of stay in an intensive care unit (10.78 days [SD 11.55] vs. 6.52 days [SD 7.45], *p* = 0.146) and overall hospital stay (17.83 days [SD 25.27] vs. 10.97 days [SD 13.81], *p* = 0.274), although neither of these reached significance in this sample. A greater percentage of patients with a spot sign underwent surgical intervention (66.67% vs. 15.52%, *p* < 0.001).

**Fig. 3:**
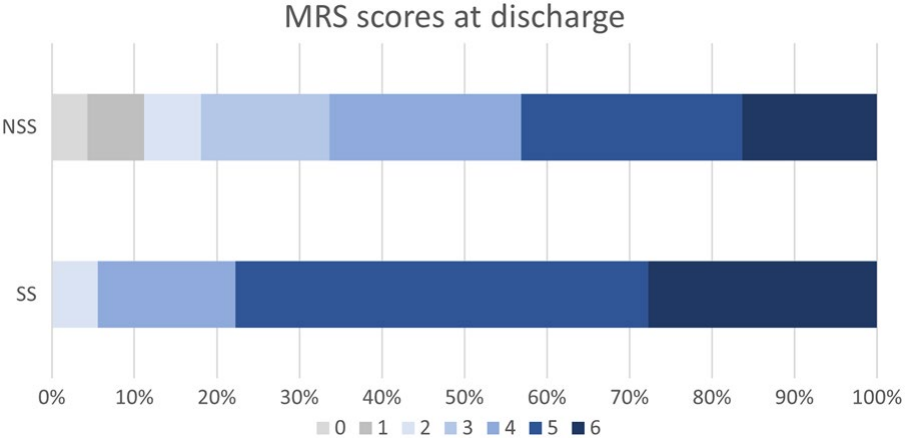
Breakdown of MRS scores for patients with a spot sign (SS) and those without a spot sign (NSS) showing the percent of each patient population with each MRS score at time of discharge. Note the greater percentage of MRS 5 and 6 in the SS population. *MRS* modified Rankin Score

Functional outcomes at time of discharge were significantly worse in patients with a spot sign compared with those without, reflected both in the GOS (2.33 [SD 0.91] vs. 3.06 [SD 1.19], *p* = 0.005) and MRS (4.94 [SD 1.00] vs. 3.92 [SD 1.64], *p* < 0.001). Analysis of MRS at time of discharge (Fig. 3) shows that this difference is driven by a greater percent of patients with a spot sign having MRS of 5 or 6 at discharge, correlating to severe disability and death, with only one patient with a spot sign having an MRS less than 4.

Death during hospitalization was not statistically different between the groups (27.78% vs. 16.38%, *p* = 0.241). The cause of death during hospitalization was the withdrawal of care in all cases except one patient with a spot sign who progressed to brain death and one patient without a spot sign who died of cardiac arrest.

## Discussion

Consistent with prior investigations, we found that a spot sign is associated with larger hematoma volumes, higher ICH scores, lower GCS on arrival, and higher rates of hematoma expansion. Although follow up is limited to the time of discharge, this study also reiterates the correlation with worse functional outcomes. We saw an increased need for operative intervention and a trend toward increased lengths of stay, indicating greater resource requirements in these patients.

Although many ICH are attributed to hypertension [29], there is poor consensus in the literature regarding elevated BP and the presence of a spot sign, with a correlation seen in some investigations [8, 13, 15, 24, 32, 33] but not in others [7, 16, 17]. Much of the ambiguity is because of variable methodologies, with some studies recording SBP and others recording MAP and inconsistent reporting of the time of BP recording in relation to the time of CTA. This study is the first, that we know of, to specifically address this question and attempt to clarify these relationships.

A significant correlation was seen in this series with elevated BP and the incidence of a spot sign, with strongest correlation at the time of CTA. This suggests a spot sign may reflect extravasation driven by elevated BP at the time of imaging. The fact that SBP has a stronger overall correlation than MAP may reflect the importance of transmural pressure in this process. Because spot sign is also associated with hematoma expansion and worsened outcomes, important questions are raised about the role of BP management in the setting of acute ICH in this population, part of a larger topic that remains controversial despite a growing literature [25].

There have been two large clinical trials investigating the impact of BP control on functional outcomes. The Antihypertensive Treatment of Acute Cerebral Hemorrhage II study [34] showed no detriment to rapid BP control, however also failed to establish benefit for hematoma expansion or functional outcomes. INTERACT-2 [26] showed no significant difference in mortality or disability at 90 days; however, an ordinal analysis of modified Rankin scores showed improved functional outcomes with intensive BP lowering. Subsequent pooled analysis of these trials also indicates that achieving early and stable SBP control was associated with favorable outcomes in ICH of mild-to-moderate severity [35].

The question is likely complicated by the heterogeneity of patients with hypertensive ICH, and certain groups require specific consideration. Particularly, it has been seen that unique caution is warranted against intensive BP lowering in patients presenting with SBP > 220 mm Hg [36] or renal impairment [34, 37].

An important confounder in this assessment is the time interval between LKW or ED arrival and spot sign on CTA. Although we saw significance of the SBP at time of CTA in a univariate model for the subset of patients with this information available, we did not see this in the multivariate model including time interval from LKW to CTA, indicating a possible impact of this variable. However, our data showed that patients with a spot sign had shorter time intervals between ED arrival and CTA (23.06 ± 32.02 vs. 176.76 ± 310.25 min, *p* < 0.001) and between LKW to CTA (133.62 ± 110.15 vs. 1578.73 ± 4188.76 min, *p* = 0.002). Because patients with spot signs had higher ICH volume, lower GCS, and higher ICH score (Table 2), it is probable they are more likely to seek medical attention immediately. In this sample, we saw a significant correlation between higher ICH score and shorter duration from arrival to obtaining a CTA by linear regression (*p* = 0.007). The time interval between LKW or ED arrival and CTA is therefore more likely a confounding factor rather than an independent risk of a spot sign.

This study suggests that one potential component of the hematoma stabilization process is the reduction in BP. That spot sign is more common in vessel imaging acquired in patients whose BP is not yet controlled or are earlier in their course of BP lowering also suggest that, rather than representing vascular pathology such as dissections or pseudoaneurysms, the spot sign more likely represents extravasation from a ruptured arterial vessel, driven by the transmural pressure.

If a spot sign represents contrast extravasation driven by elevated BP, then this may be a modifiable risk factor for hematoma expansion in these patients, which raises the question of whether this subgroup may benefit from early intensive BP lowering. The Spot Sign Score in Restricting ICH Growth study [7], a nested analysis of the Antihypertensive Treatment of Acute Cerebral Hemorrhage II study looking at patients with a spot sign, did not see significant reduction of ICH expansion with intensive BP lowering. However, this analysis groups together all patients who underwent CTA any time within the first 8 h of symptom onset [7]. It has previously been shown that a spot sign is more common with CTA sooner after symptom onset [8, 38], and the present study showed an average time from arrival to CTA of 23.06 min in patients with a spot sign, and 176.76 min in those without, both well within 8 h; however, arrival time and time of symptom onset are two very different time points and the significance of this remains unclear. It should also be noted that the present study establishes a correlation between spot sign and the SBP at time of CTA, but not on arrival, and it is not clear if the Spot Sign Score in Restricting ICH Growth study conclusions would have held if SBP at CTA were assessed. The question of optimal BP management in acute ICH remains unanswered, and while caution is warranted particularly in patients with initial SBP > 220 mm Hg or renal impairment [36, 37], patients with a spot sign are another subgroup worthy of unique evaluation in future investigations.

### Limitations

This study has several limitations. Our strict exclusion criteria may limit generalizability of these findings to other clinical presentations, including cortically based and infratentorial hematomas, which are commonly included in general ICH literature.

The assessment of outcomes in this study was limited to functional status at time of discharge, without longer term assessment. This is because of the very low rate of follow up, attributed in part to geographic considerations, as a large part of the patient population consisted of tourists visiting local attractions, patients from other countries visiting local family, and patients with insurance network preventing follow up with our center. The assessment of outcomes is also confounded by the association of spot sign with worse GCS on admission, larger hematomas, and higher overall ICH scores, which are independently associated with outcome [12, 17, 21, 22, 39], and the trial design is not appropriate to comment on surgical intervention or outcomes.

This study was performed at a tertiary academic hospital with a level 1 stroke center certification and high volume of patients with ICH, and as such the generalizability of these results to other settings such as community hospitals or centers without rigorous stroke protocols is unclear.

## Conclusions

The finding of a spot sign on CTA is significantly correlated with SBP at time of imaging and the highest SBP prior to CTA and may therefore indicate extravasation driven by uncontrolled hypertension. Spot sign is also correlated with increased severity of initial clinical presentation as well as increased operative intervention, increased mortality, and worse functional outcomes. These findings may support early intensive BP lowering in patients with a spot sign and acute ICH. The role of BP control in patients with a spot sign is a subject that deserves future investigation and clarification.
